# Knowledge attitude practice among oncologists and health care workers during COVID19 pandemic

**DOI:** 10.1186/s43046-024-00231-5

**Published:** 2024-09-09

**Authors:** Sharehan Hassan Soliman, Mahinour Mohamed Atef

**Affiliations:** Clinical Oncology and Nuclear Medicine Department, Faculty of Medicine, Suez Canal University, Ismailia, Egypt

**Keywords:** COVID-19, Oncologists, Health care workers, Knowledge

## Abstract

**Introduction:**

Healthcare providers should be well prepared to fight the COVID-19 pandemic and protect their patients and themselves as frontline workers. The aim of this study was to assess oncologists**’** and health care workers (HCWs) knowledge, attitude, and practice in response to the COVID-19 pandemic and its impact on them.

**Material and methods:**

This cross-sectional study was conducted among Egyptian oncologists and HCWs in the oncology department at Suez Canal University Hospitals, Egypt. Participants were reached through a Google Form questionnaire. The questionnaire was shared on social media (Facebook, Twitter, and WhatsApp) over four months, from June 1^st^ to September 30, 2022. All physicians and HCWs in the oncology department were invited to participate in the survey. Researchers intended to enroll all physicians and HCWs within the study period.

**Results:**

Out of the 110 participants included in the study, there was a female predominance, and the majority were oncology nurses and clinical oncologists**.** Knowledge with significant participants’ characteristics showed that knowledge significantly varied by age. The level of knowledge was significantly higher among participants between 30 and 40 years old (OR = 5.111; 95% CI, 1.202–21.738; *P* = 0.027). 65.5% of the participants had poor knowledge, with a mean ± SD of 4.9 ± 1.4. About 43.6% of the participants experienced more burnout than before the COVID-19 pandemic, with a negative emotional impact. 63.7% reported a negative financial impact due to the pandemic. 62.7% had support from their family, even though their job increases their risk of infection. 7.3% only reported a positive impact regarding their friend’s relationship.

**Conclusion:**

COVID-19 pandemic has a negative impact on oncologists' personal and professional lives. Interventions should be implemented to lessen the negative impact and better prepare oncologists to handle future crises with greater efficiency and resilience.

## Introduction

COVID-19, which emerged at the end of 2019 and spread rapidly over the world, was designated a pandemic by the WHO on March 11, 2020 [14].

This epidemic has overloaded healthcare systems in many nations, causing interruptions in care and a lack of resources, negatively impacting care delivery for COVID-19 patients and others [15].

Coronavirus disease 2019 (COVID-19) has generated a worldwide health catastrophe and has startled HCWs because of its wide, dynamic, and varied clinical presentation, ranging from asymptomatic to severe illness resulting in hospitalization and death.

The fast development of accessible material and the daily emergence of new information on the pandemic have made it difficult for HCWs to keep current with the newest knowledge, which may impact their attitudes and behaviors during patient care [4].

Furthermore, HCWs are at a significant risk of contracting the virus and serving as a source of transmission in the community. Previous research found that HCWs lacked knowledge and attitudes concerning COVID-19 [12].

Doctors confront enormous challenges as they are required to comprehend and apply all newly acquired facts about the pandemic in order to give better clinical treatment and safeguard themselves and their patients from damage [13].

In the pandemic, oncologists face the same challenges as previously mentioned, including protecting their patients, coworkers, themselves, and their families while continuing to provide timely care to their patients in suboptimal circumstances due to overburdened healthcare systems and a lack of clear evidence on appropriate action [11].

While the majority of prior research analyzing COVID-19’s influence on HCWs was conducted in the Western world, nothing has been published in poor and low-middle-income countries (LMICs). In light of this, we undertook this study to analyze Egyptian oncologists’ and HCWs responses to the pandemic in terms of knowledge, attitude, and behaviors, as well as to assess the pandemic's influence on many parts of their lives, especially in our institute. Also, to increase the level of awareness and health precautions among the medical team, besides governmental and health authorities' guidelines to prevent transmission.

Our oncologists and HCWs help cancer patients cope with the COVID-19 pandemic. Cancer Patients were reassured and instructed to decrease the number of follow-up visits. Many intravenous drugs were converted to oral systemic therapy. Radiotherapy was also given with the least fractionation allowed according to treatment protocols. Hospital admission was restricted to emergency situations, and certain selected maintenance therapies were allowed with reduced frequency.

Patients were encouraged to cope with stress, including physical activity, a balanced diet, adequate sleeping hours, avoiding overload of information, and social stress.

Telemedicine is an excellent and successful experience [7]. Telemedicine allows the exchange of medical information between our medical team and patients using electronic communication. Telemedicine proved to be a positive experience that provided access to medical care. Communication with cancer patients provides psychological support, medical advice, convenience, reduced costs, and a decrease in the rate of unneeded hospital admissions.

## Subject and methods

### Study design and participants

This cross-sectional study was conducted among Egyptian oncologists and HCWs in the oncology department at Suez Canal University Hospitals, Egypt. Participants were reached through a Google Form questionnaire. The questionnaire was shared on social media (Facebook, Twitter, and WhatsApp) over four months, from June 1st to September 30, 2022. All physicians and HCWs in the oncology department were invited to participate in the survey. Researchers intended to enroll all physicians and HCWs within the study period.

### Procedures

Demographic data, knowledge, attitude, behavior, belief, awareness, and impact data were collected through a Google Form questionnaire. Target populations include oncologists (clinical, medical, surgical, pediatric, hemato-oncologists), and HCWs. HCWs include clinical pharmacists, pathologists, physicists, radiotherapy technicians, and oncology nurses.

An invitational message with a link to a Google Form questionnaire was sent to study participants. The questionnaire contained 37 questions, and data collection was done over four consecutive months, from June 1^st^ to September 30, 2022.

The questionnaire contains four main domains: (1) Participants' characteristics include age, gender, specialty, years of experience, and site of work. (2) Knowledge about COVID-19; (3) Participant’s behavior; (4) Safety at the workplace and Participant’s attitude towards COVID-19 viral infection; (5) Participant’s beliefs.

The KAP survey was designed based on the previous data published by the WHO and the Center for Disease Control and Prevention. Participant’s knowledge was assessed by nine questions. Each question was given a score of 1 for the correct answer and 0 for the incorrect answer. The questions included the participants' knowledge about clinical presentations (1 item), transmission route (1 item), prevention (6 items), treatment, and control (1 item) of COVID-19 infection.

Safety measures and participants' attitudes were assessed by five questions to assess participants’ attitudes towards COVID-19. Participants were asked about their opinions towards contacting COVID-19-infected patients and colleagues and their degree of satisfaction with their workplace instructions to prevent transmission and their management plan.

To assess participants' beliefs, participants were asked 10 questions. Questionnaires include participants' beliefs about the risk of infection at work, how they feel about their work, shortages of manpower and facilities, access to COVID-19 diagnostic measures and treatment in cases of infection, and the safety and efficacy of the COVID-19 vaccine.

Mental and emotional well-being and financial impact were assessed by six questions. Participants were asked (1) whether they experienced more burnout than before the pandemic, (2) whether their financial resources decreased, (3) whether the pandemic affected their plan of management, (4) whether they got infected and the source of infection according to their viewpoint, and (5) whether they got family or friend support.

The questionnaire aims to assess the level of knowledge about COVID-19 pandemic and protective measures, also the pandemic emotional, financial, and psychological impact on the study participants. Questionnaire included participants’ attitude towards COVID-19 vaccine and patients’ compliance to new treatment instructions and the impact of that on their plan of management.

### Data analysis

Assessment of knowledge towards COVID-19 was done via google form questionnaire. Each correct answer was given a point. The total score of knowledge ranged from 0 to 9. Participants with score from 0 to 5 were evaluated as poor knowledge and those with score from 6 to 9 were evaluated as good knowledge.

The χ2 test of independence was done to assess the variation of study variables across the level of knowledge. Odds ratios (ORs) and 95% CIs (confidence intervals) were calculated using logistic regression analysis to ascertain the effects of study variables on the likelihood of having poor knowledge towards COVID-19 and intentions to take the COVID-19 vaccine if it becomes available. The significance level was set at *P* value less than 0.05.

### Ethical approval

All eligible participants were informed about the aims & objectives of the study, and they agreed for participation. This study was approved by research ethics committee of Faculty of Medicine Suez Canal University.

## Results

Out of the 110 participants included in the study, there was a female predominance, and the majority were oncology nurses and clinical oncologists. Table 1 includes the participant’s characteristics.

**Table 1:** Participant’s characteristics & relation between knowledge and personal characteristic (*n* = 110)

Characteristic	Total (*n* = 110)		Knowledge				χ^2^	p
			**Good (*n* = 38)**		**Poor (*n* = 72)**			
	**No**	**%**	**No**	**%**	**No**	**%**		
**Age (/years)**								
Less than 30	33	30.0	10	30.3	23	69.7	6.126^*^	0.047^*^
30 – 40	67	60.9	21	31.3	46	68.7		
40 – 50	10	9.1	7	70.0	3	30.0		
**Gender**								
Male	26	23.6	8	30.8	18	69.2	0.215	0.643
Female	84	76.4	30	35.7	54	64.3		
**Specialty**								
Clinical Oncologist	28	25.5	8	28.6	20	71.4	3.776	^MC^*p* = 0.911
Medical Oncologist	12	10.9	4	33.3	8	66.7		
Radiation Oncologist	8	7.3	4	50.0	4	50.0		
Surgical Oncologist	4	3.6	2	50.0	2	50.0		
Clinical Pharmacist	4	3.6	2	50.0	2	50.0		
Oncology nurse	38	34.5	12	31.6	26	68.4		
Physicist	4	3.6	1	25.0	3	75.0		
Radiotherapy Technician	6	5.5	2	33.3	4	66.7		
Pathologist	6	5.5	3	50.0	3	50.0		
**Years of experience**								
Less than 5	27	24.5	10	37.0	17	63.0	2.309	0.679
5 to 10	34	30.9	10	29.4	24	70.6		
11 to 15	25	22.7	8	32.0	17	68.0		
16 to 20	15	13.6	5	33.3	10	66.7		
More than 20	9	8.2	5	55.6	4	44.4		
**Site of work**								
University hospital	85	77.3	29	34.1	56	65.9	7.133	^MC^*p* = 0.068
Private hospital	8	7.3	1	12.5	7	87.5		
Health insurance	4	3.6	0	0.0	4	100.0		
Ministry of health hospitals	13	11.8	8	61.5	5	38.5		

*χ*^*2*^ Chi square test, *MC* Monte Carlo

*p* *p* value for comparing between Good Knowledge and Poor Knowledge

^*^Statistically significant at *p* ≤ 0.05

### Participant’s knowledge

Participant’s knowledge was assessed by nine questions. Each question was given a score of 1 for the correct answer and 0 for the incorrect answer. 65.5% of the participants had poor knowledge, with a mean ± SD of 4.9 ± 1.4 (Table 2).

**Table 2:** Distribution of the studied cases according to assessment of knowledge & behavior: (*n* = 110)

Q	Assessment of knowledge	Incorrect	Correct
		**No. (%)**	**No. (%)**
**1**	**Do you think that only old immunocompromised patients are at risk of severe corona virus infection with hospital admission?**	65 (59.1%)	45 (40.9%)
**2**	**Do you think that COVID 19 viral infection is transmitted via respiratory droplets of infected individuals?**	17 (15.5%)	93 (84.5%)
**3**	**I can be a virus carrier even if I don’t have symptoms of fever or cough**	22 (20.0%)	88 (80.0%)
**4**	**You need to wear a surgical mask all the times while at work to avoid getting infected with COVID-19**	97 (88.2%)	13 (11.8%)
**5**	**You need to wear N95 mask when I am examining my cancer patients to avoid getting infected with COVID-19**	63 (57.3%)	47 (42.7%)
**6**	**I should stay home, isolate myself, and inform my supervisor if I have fever or cough even if I don’t have known exposure to COVID-19 patient or recent travel**	22 (20.0%)	88 (80.0%)
	Correct answer: Yes		
**7**	**I should apply social distancing outside work**	71 (64.5%)	39 (35.5%)
	Correct answer: Yes		
**8**	**Shaking hands with others**	93 (84.5%)	17 (15.5%)
	Correct answer: No		
**9**	**More aware about hand washing**	4 (3.6%)	106 (96.4%)
	Correct answer: Yes		
	**Total knowledge**		
	Good knowledge	38	34.5
	Poor knowledge	72	65.5
	**Total score**		
	Mean ± SD	4.9 ± 1.4	
	Median (Min. – Max.)	5 (0 – 8)	

*SD* Standard deviation

Univariate logistic regression analysis for knowledge with significant participants’ characteristics showed that knowledge significantly varied by age. Level of knowledge was significantly higher among participants between 30 and 40 years old (OR = 5.111; 95% CI, 1.202–21.738; *P* = 0.027), followed by participants less than 30 years of age (OR = 5.367; 95% CI, 1.147–25.105; *P* = 0.033).

About 59% of the participants thought that only old immunocompromised patients were at risk of developing severe COVID-19 viral infection with hospital admission. 84.5% answered correctly that the COVID-19 viral infection is transmitted via respiratory droplets in infected individuals. 80% of the participants answered correctly that they could be a viral carrier even though they did not have symptoms.

88.2% of the participants thought that they needed to wear a surgical mask all the time while at work to avoid getting infected with COVID-19, even if they did not take care of patients. 57.3% thought they needed to wear an N95 mask while examining cancer patients to avoid getting infected with COVID-19.

### Participant’s behavior

Adherence to precautionary measures against the COVID-19 viral infection, such as social distancing, handshaking, and hand washing, was variable among participants, with hand washing being the highest compliance practice. 64.5% of the participants strictly apply social distancing outside work [OR = 0.882, 95% CI: (0.397–1.959), *P* value = 0.758]. 84.5% shake hands with others [OR = 2.347, 95% CI: (0.711–7.346), *P* value = 0.161]. 96.4% became more aware of hand washing [OR = 1.585, 95% CI: (0.215–11.698), *P* value = 0.651].

### Safety at the workplace and the participant’s attitude towards the COVID-19 viral infection

Safety measures and participant attitudes were assessed by five questions. Each answer was given a score with a cutoff point of 0–4 for poor safety measures and 5–8 for good safety measures. 60.9% of the participants thought they had good safety measures at the workplace and felt satisfied (mean ± SD 4.99 ± 1.4).

About protection against COVID-19 infection, 54.5% of the participants did not feel satisfied with the hospital measures. 65.5% did not feel comfortable treating infected COVID-19 cancer patients. 92.7% of the participants worked with a previously infected COVID-19 colleague. 49.1% of the participants thought it would be better to work with a proven uninfected colleague without any symptoms, whereas 40.9% would work with their colleagues regardless of their infectious status due to good safety measures (Table 3).

**Table 3:** Distribution of the studied cases according to attitude towards COVID-19 (*n* = 110)

Q	Attitude towards COVID-19	No. (%)
**10**	**About protection against COVID-19 at your workplace, do you think?**	
	I am well protected against Covid-19 viral infection^a^ (score 2)	29 (26.4%)
	I am not protected against Covid-19 viral infection (score 0)	60 (54.5%)
	I don't Know (score 1)	21 (19.1%)
**11**	**Have you ever contacted a COVID-19 cancer patient?**	
	Yes^a^ (score 1)	77 (70%)
	No (score 0)	33 (30%)
**12**	**Do you feel comfortable treating infected Covid 19 cancer patients?**	
	Yes^a^ (score 1)	38 (34.5%)
	No (score 0)	72 (65.5%)
**13**	**Have you ever worked with a previously COVID-19 infected colleague?**	
	Yes^a^ (score 1)	102 (92.7%)
	No (score 0)	8 (7.3%)
**14**	**In your opinion, which would be better**	
	I feel comfortable to work with my colleagues who are currently not infected (score 2)	54 (49.1%)
	I will only work with a colleague who never had a history of COVID-19 infection (score 0)	1 (0.9%)
	I will only work with a colleague who are sure to be not infected or have symptoms (score 1)	10 (9.1%)
	Its ok to work with my colleagues regardless of the infection status* (score 3)	45 (40.9%)
	**Total score**	
	Poor safety measures (0 – 4)	43 (39.1%)
	Good safety measures (5 – 8)	67 (60.9%)
	**––––––––––––––––––––––––-**	
	Mean ± SD	4.99 ± 1.4
	Median (Min. – Max.)	5 (2 – 8)

*SD* Standard deviation

^a^Right answer

### Participant’s beliefs

About 41.8% thought it was easy and available to do PCR at their institute. 63.6% were worried that their job put them at increased risk of infection, even though they would continue to work. 86.4% of the participants received the COVID-19 vaccine. Only 12.7% of them will receive the vaccine regularly. 54.5% of the participants stated that they would not pay money to receive the vaccine. 27.3% of the participants thought that hemato-oncology was the most affected oncology branch by the pandemic, while 33.6% thought that radiation oncology was the least affected oncology branch (Table 4).

**Table 4:** Distribution of the studied cases according to beliefs about COVID-19 (*n* = 110)

Q	Beliefs about COVID-19	No. (%)
**15**	**Do you think it's easy and available to do PCR test in case you are infected?**	
	Yes*	46 (41.8%)
	No	42 (38.2%)
	I don’t know	22 (20%)
**16**	**What about your feeling about your job?**	
	Same feeling as before the pandemic*	36 (32.7%)
	Feeling that job puts them at an increased risk but will continue in it	70 (63.6%)
	I will change my job in next few years	4 (3.6%)
**17**	**Have you received a Covid 19 vaccine?**	
	Yes*	95 (86.4%)
	No	15 (13.6%)
**18**	**Would you get the flu vaccines**	
	Yearly*	14 (12.7%)
	Occasionally	44 (40%)
	Not at all	52 (47.3%)
**19**	**Would you pay money to receive the vaccine?**	
	Yes*	14 (12.7%)
	No	60 (54.5%)
	May be	36 (32.7%)
**20**	**Do your colleagues suffer from shortage in manpower and facilities in covid-19 pandemic?**	
	Always	34 (30.9%)
	Sometimes	60 (54.5%)
	Rarely	9 (8.2%)
	Never*	7 (6.4%)
**21**	**Do patient accept new instructions and guidelines in Covid era?**	
	Always*	20 (18.2%)
	Sometimes	81 (73.6%)
	Rarely	8 (7.3%)
	Never	1 (0.9%)
**22**	**Do patient lie about symptoms and deny infection?**	
	Always	14 (12.7%)
	Sometimes	79 (71.8%)
	Rarely	10 (9.1%)
	Never*	7 (6.4%)
**23**	**In your opinion, which oncology branch is mostly affected by Covid-19 pandemic?**	
	Radiation oncology	15 (13.6%)
	Medical Oncology	33 (30%)
	Pediatric Oncology	5 (4.5%)
	Surgical Oncology	8 (7.3%)
	Palliative Care Unit	19 (17.3%)
	Haemato-Oncology	30 (27.3%)
**24**	**In your opinion, which oncology branch is Least affected by Covid-19 pandemic?**	
	Radiation oncology	37 (33.6%)
	Medical Oncology	11 (10%)
	Pediatric Oncology	11 (10%)
	Surgical Oncology	23 (20.9%)
	Palliative Care Unit	23 (20.9%)
	Hemato-Oncology	5 (4.5%)

*Health care workers experienced different levels of financial & psychological impact during the pandemic. It was important to determine how they feel about their job and their access to the vaccine

### Impact of Participants’ Well-Being and Social, Financial, and Professional Life

About 43.6% of the participants experienced more burnout than before the COVID-19 pandemic, with a negative emotional impact. 63.7% reported a negative financial impact due to the pandemic. 62.7% had support from their family, even though their job increases their risk of infection. 7.3% only reported a positive impact regarding their friend’s relationship. 49.1% of the participants stated that COVID-19 pandemics affect their plans of management and expectations for cancer patients.

Mental, emotional well-being, and financial impact varied by age, with participants less than 30 years of age being more likely to be affected (OR = 0.073, 95% CI 0.007 to 0.809, *P* value 0.033) (Table 5).

**Table 5:** Distribution of the studied cases according to impact of COVID-19 on wellbeing and social life (stigma) (*n* = 110)

Q	Impact of COVID-19 on wellbeing and social life (stigma)	No. (%)
**25**	**Do you experience more burnout than before the COVID-19 pandemic? (Mental and emotional wellbeing)**	
	Yes … (negative impact)	48 (43.6%)
	No … (positive impact*)	26 (23.6%)
	Maybe … (no change)	36 (32.7%)
**26**	**Did you experience decrease in your financial resources due to Covid-19 pandemic? (financial impact)**	
	My income was not affected. (positive impact*)	40 (36.3%)
	Affected by less than 20%… (negative impact)	32 (29.1%)
	Affected less than 50%… (negative impact)	20 (18.2%)
	Affected more than 50%… (negative impact)	18 (16.4%)
	My income increase… (positive impact*)	0 (0%)
**27**	**Does Covid 19 pandemics affect you plan of management & expectations for cancer patients?**	
	Yes	54 (49.1%)
	No	17 (15.5%)
	Maybe	39 (35.5%)
**28**	**Have you ever got corona virus infection?**	
	Yes	66 (60%)
	No	21 (19.1%)
	I do not know	23 (20.9%)
**29**	Do you have support from your family and friends or they reduce their contact with you?* (family and friend relationship)	
	My family always support me, even that my job increase their risk of infection (positive impact)*	69 (62.7%)
	My friends reduce their contact with me (negative impact)	12 (10.9%)
	My family did not feel comfortable with my job (negative impact)	21 (19.1%)
	My friends always support me and did not isolate themselves from me (positive impact)	8 (7.3%)
**30**	**From where you think you got corona virus infection?**	
	Cancer patient	6 (5.5%)
	Colleague	8 (7.3%)
	Family member	10 (9.1%)
	Friend or neighbor	1 (0.9%)
	I don't know from where I got my infection	60 (54.5%)
	I did not get Covid-19 virus infection before	25 (22.7%)

*Health care workers experienced different levels of financial & psychological impact during the pandemic. It was important to determine how they feel about their job and their access to the vaccine

## Discussion

Our study comprised oncologists and HCWs at various stages of their careers and experiences. The study yielded intriguing findings on knowledge habits and the influence of the pandemic on participants.

A high proportion of participants had a low level of knowledge, which represents about 65.5%. In our study, 59% of participants suspected that old immune compromised patients were at risk of COVID, and 88.2% needed to wear surgical masks all the time during work. While 57.3% thought they needed to wear an N95 mask while examining cancer patients.

This goes with the study done on Middle East and North African countries, which may reflect the nature of the pandemic with numerous uncertainties, depending extensively on social media and other nonprofessional sources of information, and a varying COVID-19 effect in different countries [9].

This entails ensuring genuine and accessible sources of accurate information that people can rely on, rather than relying on social media and other untrustworthy sources. This is especially true for healthcare practitioners, since information influences attitudes, health habits, and practices [5, 16, 17].

In addition, hand washing was the highest compliance practice among participants, with 96.4% becoming aware of hand washing, whereas hand shaking is a less likely compliance practice.

About participants attitudes towards the COVID viral infection 65.5% are worried about treating COVID cancer patients and feel less comfortable, and about 40.9% would work with their colleagues regardless of their infectious status due to good safety measures.

Aside from the fear and poor impact on mental well-being, our study indicated that the pandemic had a diverse influence on participants, including their relationships with family and coworkers, financial income, and research productivity.

In our study, 43.6% of the participants had a negative emotional impact due to more burnout than before the COVID-19 pandemic, and 63.7% had a negative financial impact. The plan of management and expectations for cancer patients affected by COVID. There is a positive impact regarding family support.

In a Brazilian survey of 744 urologists, 54% reported a 50% decrease in their income [6]. Another study indicated that imaging investigations will be reduced by 50%–70% for 3–4 months [2].

Previous research has also reported on the influence of COVID-19 on scientific pursuits. More than 80% of the 165 breast oncologists polled in Italy indicated a reduction in their scientific and research activity [10].

However, several individuals reported a beneficial influence of the epidemic on many factors that were studied, such as relationships with family, coworkers, and research productivity.

In another study, oncologists in Brazil experienced the most negative impact on their mental well-being and relationships with family and coworkers, as well as a negative influence on their financial position and income. They were, however, more likely to show a desire to take the COVID-19 vaccination whenever it became available. The causes of regional disparities in knowledge and some of the participants' behaviors need to be investigated further, although they can be related to the entire country's experience with the epidemic, accessible information sources, or cultural heterogeneity [1, 3, 8].

## Conclusion

Our research emphasized the multifaceted influence of the COVID-19 pandemic on numerous areas of oncologists and HCWs personal and professional lives. These findings need the implementation of treatments that ensure oncologists and HCWs well-being and productivity.

### Study limitations

The limitation of our study is related to the cross-sectional design, which captures information at a specific point in time and may change over time. This design was rapidly evolving and introducing new knowledge and facts that may affect physicians' behaviors and feelings.

## Data Availability

The data that supports the findings of this study is available from the corresponding author upon reasonable request.

## Abbreviations

CIs: Confidence intervals
COVID-19: Coronavirus disease 2019
FOMSCU: Faculty of medicine at Suez Canal University
HCWs: Health care workers
LMICs: Low- and middle-income countries
ORs: Odds ratios
